# Prevalence of Comorbidities among Patients with Acromegaly

**DOI:** 10.12669/pjms.37.7.4277

**Published:** 2021

**Authors:** Sajjad Ali Khan, Nanik Ram, Muhammad Qamar Masood, Najmul Islam

**Affiliations:** 1 Dr. Sajjad Ali Khan, FCPS. Department of Medicine, Section of Endocrinology, Aga Khan University Hospital, Karachi, Pakistan; 2 Dr. Nanik Ram, FCPS. Department of Medicine, Section of Endocrinology, Aga Khan University Hospital, Karachi, Pakistan; 3 Dr. Muhammad Qamar Masood, MD. Department of Medicine, Section of Endocrinology, Aga Khan University Hospital, Karachi, Pakistan; 4 Dr. Najmul Islam, FRCP. Department of Medicine, Section of Endocrinology, Aga Khan University Hospital, Karachi, Pakistan

**Keywords:** Acromegaly, Complications, Comorbidities

## Abstract

**Background and Objective:**

Acromegaly is a chronic disorder resulting from excessive secretion of growth hormone and (GH) and insulin-like growth factor 1 (IGF-1) and is associated with several comorbidities. These complications contribute significantly to morbidity and mortality associated with this condition thus early diagnosis leads to better outcomes. There have been studies in other countries to assess the comorbidities associated with acromegaly. However, we do not have any recent data with regards to Pakistan. So, in order to demonstrate the prevalence of demographics, hormonal disorders, and other complications associated with acromegaly we conducted this study.

**Methods:**

It is a retrospective review of patients’ records presented to the tertiary care Hospital, Karachi, Pakistan for the diagnosis and management of acromegaly and the complications associated with this condition between the time periods 2000 till 2020. A total of 89 patients fulfilled the inclusion criteria of acromegaly and were included in the study. Comorbid conditions were described based on current guidelines. Patient baseline characteristics were recorded along with other complications arising during treatment.

**Results:**

Eighty-nine patients were included. 64% were male, over 70% were older than 30 years old and more than 40% of patients had BMI greater than 30. HTN, pre-hypertension, and CCF were reported in 35.95%, 3.37%, and 6.74%. Diabetes mellitus, hypocortisolism, hypothyroidism, hypogonadism, and hyperprolactinemia were reported in 39.32%, 38.20%, 37.07%, 34.46%, and 16.85% of cases. The prevalence of osteoarthritis, blood disorder, skin changes, thyroid cancer, and spinal stenosis was found out to be around 1.12% each.

**Conclusions:**

Acromegaly is associated with cardiovascular and endocrinal disorders. Screening for these disorders at the time of diagnosis can lead to early management and better outcomes translating into decreased mortality.

## INTRODUCTION

Acromegaly is a chronic progressive disease caused by an excess secretion of growth hormone from the pituitary adenomas and the resultant increase in an insulin-like growth factor (IGF-1). Chronic exposure to high levels of GH leads to a variety of complications. It has been postulated that it is associated with two to three time’s higher mortality than the general population.

The mortality is related to an excess of cardiovascular (hypertension, heart failure), cerebrovascular (stroke), and respiratory disorders (obstructive sleep apnea). Moreover, studies have reported a higher prevalence of malignancies, osteoarthritic changes, endocrinological and metabolic disorders in the form of hyperglycemia, lipid disorders to name a few. As a result of these morbidities, the mortality rate is increased in these patients.^1^

It has been recommended to diagnose these disorders earlier on before they become irreversible. Among all the complications, cardiovascular disorders account for a greater than two-third of morbidities and around half of the mortality.^2^ Among the cardiovascular disorders, studies have reported cardiomyopathies, ischemic heart disease, systemic arterial hypertension, rhythm disorders, and valvular lesions in various frequencies. Among these cardiomyopathies, leading to heart failure and systemic hypertension are much more prevalent than others.^3^,^4^ Respiratory disorders in the form of obstructive sleep apnea and respiratory insufficiency can lead to morbidities in these patients.^5^-^7^ Among the metabolic complications, studies have reported varying frequencies of dysglycemia, impaired fasting glucose, and impaired glucose tolerance.^8^,^9^

Our objective was to demonstrate the prevalence of demographics, hormonal disorders, and other complications associated with acromegaly

## METHODS

It was a retrospective review of patients’ records presented to the tertiary care Hospital, Karachi, Pakistan for the diagnosis and management of acromegaly. After the approval from the ethical review committee (ERC) of our center, we requested the health information and management services (HIMS) to provide the list of patients diagnosed to have pituitary tumors.

A total of 89 patients fulfilled the diagnosis of acromegaly based on the clinical characteristics and biochemical markers and were included in the study. These patients were either undergoing treatment with surgery/ medical therapy for acromegaly or on being followed up in a tertiary care hospital, Karachi, Pakistan.

The inclusion criteria included confirmed cases of acromegaly fulfilling the clinical features and endocrine society diagnostic criteria, i.e. 1) patients with elevated or equivocal serum IGF-1 levels matched for age and sex, and confirmation of the diagnosis by finding lack of suppression of GH to < 1 μg/L following documented hyperglycemia during an oral glucose load.^10^

Patients aged 15 years or below were also excluded from the study, as body mass index (BMI) and blood pressure vary in childhood. Data was collected retrospectively by reviewing the patient records for both the baseline and subsequent visit post-intervention (surgery and medical therapy).

For each patient, we analyzed the following data recorded by the primary physician at the diagnosis of acromegaly: age and sex, BMI, estimated duration since acromegaly diagnosis, mean serum GH, IGF-1 level (matched for age and sex in each center), pituitary tumor size measured by magnetic resonance imaging (MRI), and presence of, FBS, two hours glucose level (RBS) and HbA1C. Endocrine Society guidelines were used to report hypothyroidism, hypogonadism, adrenal insufficiency, and hyperprolactinemia.^11^

We reviewed the patient records and the echocardiographic findings for the diagnosis of cardiomyopathy and valvular lesions. Similarly, musculoskeletal disorders were recorded as mentioned in the records based on the radiological investigation done during the treatment of acromegaly. With regards to dysglycemia, patients were classified according to the diagnostic criteria of the American Diabetes Association (ADA).^12^

Blood pressure was classified according to the recently released Seventh Report of the Joint National Committee (JNC) on Prevention, Detection, Evaluation, and Treatment of High Blood Pressure. A systolic B.P (SBP) of 120 to 139 mm Hg or a DBP of 80 to 89 mm Hg is defined as prehypertension. Hypertension is classified using cutoff points of SBP of 140 mm Hg or higher or DBP of 90 mm Hg or higher.^13^ Patient data was then entered in excel initially and then all statistical analyses were performed using the SPSS 16.0 software.

## RESULTS

The general characteristics of the study population are shown in Table-I. The majority of the study patients were young falling in the age range between 15 to 45 years of age (77.54%) with sixty-four percent being males. Most of the patients had a BMI between 25-35 (77.51%) and were diagnosed within five years of disease onset (67.41%).

**Table I:** General characteristics of patients.

		*N (%)*
Age	15-30 years	26(29.21)
	30-45 years	43(48.31)
	>45 years	20(22.47)
Gender	Male	57(64.0)
	Female	32(35.95)
BMI	<25	12(13.43)
	25-30	33(37.07)
	30-35	36(40.44)
	>35	8(8.98)

BMI, body mass index; cm, centimetre; >, greater than; <, less than; %, Percentage.

The prevalence of cardiovascular, respiratory, musculoskeletal, skin and blood disorders is shown in |Table-II. The table shows that 3.37% (n = 3) of the patients were in a state of pre-hypertension and 35.95% (n = 32) of the patients were reported to be hypertensive. 6.74% (n = 6) of the patients had comorbid CCF, OSA was reported in a subset of patients 14.8% (n = 12).

**Table II:** Frequency of cardiovascular, respiratory, and other conditions.

*Disorder*	*N (%)*
Pre-Hypertension	3 (3.37)
Hypertension	32 (35.95)
Congestive Cardiac Failure	6 (6.74)
Infective Endocarditis	1 (1.12)
Cardiac Valvular Lesions	2(2.24)
Obstructive sleep apnea	12 (14.8%)
Osteoarthritis	1 (1.12)
Osteoporosis	2 (2.24)
Spinal Stenosis	1 (1.12)
Multinodular Goiter	2 (2.24)
Thyroid Nodule	1 (1.12)
Thyroid Cancer	1(1.12)
Colonic Polyp	2 (2.24)
Skin Tags & Gastrointestinal Bleeding & Myopathy	1 (1.12)
Deafness	1 (1.12)
Blood Disorder	1 (1.12)
Peripheral Neuropathy	4(4.49)

The frequency of various endocrine disorders in our study population are illustrated in Table-III. Our study reviewed multiple hormonal abnormalities that coexisted in patients with pituitary adenoma, including hypothyroidism, hypocortisolism, hypogonadism, and prolactinoma.

**Table III:** Endocrine related disorders in acromegaly.

*Disorder*	*N (%)*
Normoglycemics	43 (48.3%)
Diabetes Mellitus	35 (39.32)
Impaired Fasting Glucose	5 (5.61)
Impaired Fasting Glucose & Impaired Glucose Tolerance	5 (5.61)
Hypocortisolism	34(38.20)
Hypogonadism	28(31.46)
Prolactinoma	15(16.85)
Hypothyroidism	33(37.07)
Dyslipidemia	10 (12.3)
Prolactinoma	15 (16.85)
Amenorrhea	10 (31.25)
Impotence	6 (10.52)
Infertility	4 (4.49)

## DISCUSSION

For this study, we assessed 89 Patients that is after applying exclusion criteria. Males accounted for a higher proportion of our study population (64.0%). Our study found that the highest incidence of pituitary adenoma with acromegaly occurred in the middle age group 30-45-year-old (40.44%), and most people had above-normal BMI >25-30.

Our study shows a very high prevalence of diabetes 39.32 % among acromegalic patients as compared to the general population of this country 19.4 % as shown in the International Diabetes Federation Diabetes Atlas, 9^th^ edition.^14^ Furthermore, the prevalence of prediabetes (IFG, IGT) was 12.35 % among acromegalic patients.

This higher prevalence of 39.32 % is in parallel withover 50 % of prevalence reported in the Russian population reported by A V Deval et al^8^, 31.9% in the Mexican population^15^, 28% in the Belgian population. Cardiovascular complications are very common in acromegaly. The Prevalence of pre-hypertension and hypertension in our study was found to be 3.37% and 35.95% respectively which is comparable to other studies done in other countries.^16^,^17^ However, it was much higher (26.34% vs 35.95% ) than the general population as reported in a recent metanalysis.^18^ Our study reports the frequency of Congestive Cardiac Failure as a comorbid condition to be 6.67%, whereas in literature it is reported to be ranging between 1 and 4%.^19^

Similarly, our study reveals that the prevalence of valvular lesions and infective endocarditis is 2.24 and 1.12% respectively. This is inconsistent with studies reported earlier.^20^ The prevalence of OSA in our study was reported to be 12 (14.8%) in contrast other studies report a much higher prevalence of OSA ranging from 27 % in older studies to over 80 % in other studies.^21^ As it is linked to higher BMI, and generally underreported. Hypopituitarism is a known complication of acromegaly leading to secondary adrenal insufficiency, hypogonadotropic hypogonadism, and secondary hypothyroidism. In our dataset, Hormonal abnormalities were reported to be 38.20%, 31.46%, 37.07% for hypocortisolism, hypogonadism, and hypothyroidism respectively. The prevalence of hyperprolactinemia was found out to be 16.85% which is lower than the previously reported (30 %).^22^ Similarly, the prevalence of amenorrhea and infertility was found in 11.23%and 4.49% respectively which is much lower than other studies.^23^ One of the possible reasons could be the fewer number of female patients in our cohort.

Our study reports that the prevalence of osteoarthritis and spinal stenosis is 2.24%, 1.12%, and 1.12% respectively. This is in contrast to other studies that reported a very high prevalence of arthropathy of 56%,^24^ and 10.6% for vertebral fractures.^25^ Most of our patients presented earlier (< 5 years), this might be the reason for the low rates of rheumatological disorders. Studies have reported a higher prevalence of GI cancers in acromegalic patients. In contrast, our study did not find any significant association between acromegaly colon cancers. Only 2.24% of patients had colonic polyps which is much lower than reported in other studies.

## CONCLUSION

Acromegaly is associated with several comorbid conditions most importantly cardiovascular and metabolic complications. Early diagnosis can lead to a better outcome.

### Authors’ Contribution:

**NA:** Conceived, designed, and did statistical analysis.

**SAK:** Did data collection and manuscript writing.

**MQM:** Did the editing of the manuscript.

**NI:** Did a review and final approval of the manuscript.
